# Considering Community: Examining Civic Engagement and Well-Being in Later Life

**DOI:** 10.1093/geroni/igaf122.3493

**Published:** 2025-12-31

**Authors:** Lauren Gil Hayes, Morgan Stangl, Man Guo, Yi Wang

**Affiliations:** University of Iowa, Iowa City, Iowa, United States; University of Iowa, Iowa City, Iowa, United States; University of Iowa, Iowa City, Iowa, United States; University of Iowa School of Social Work, Iowa City, Iowa, United States

## Abstract

The socio-ecological model (SEM) highlights how well-being is shaped by individual, interpersonal, and community-level factors. While individual behaviors and interpersonal relationships are well-documented influences on well-being, civic engagement—through volunteer activities, adult learning, and special interest group participation—remains an underexamined community-level engagement factor. This study investigates whether civic engagement is associated with loneliness and health in aging adults and whether interpersonal support moderates these associations. Using data from the 2018 Health and Retirement Study (HRS) Leave-Behind Questionnaire (n = 5,530), a nationally representative survey of aging U.S. adults, we employ three survey-weighted Ordinary Least Squares (OLS) regressions with robust standard errors, adjusting for demographic and socioeconomic characteristics. Results indicate that higher civic engagement is significantly associated with lower loneliness (β = -0.0436, p < 0.001) and better self-rated health (β = 0.1386, p < 0.001). The magnitude of the association between civic engagement and self-rated health is larger than its association with loneliness. Interpersonal support from spouses, children, and friends does not significantly moderate these relationships, inviting further analysis to determine if civic engagement may be beneficial for those with limited support networks. These findings position civic engagement as a potential public health strategy, reinforcing and expanding SEM’s multi-dimensional emphasis on structural and social contexts in well-being. Expanding opportunities for volunteerism, lifelong learning, and group participation may enhance both social and health outcomes, reinforcing the importance of community involvement in shaping a holistic understanding of well-being.

